# Substrate Type Determines Metagenomic Profiles from Diverse Chemical Habitats

**DOI:** 10.1371/journal.pone.0025173

**Published:** 2011-09-23

**Authors:** Thomas C. Jeffries, Justin R. Seymour, Jack A. Gilbert, Elizabeth A. Dinsdale, Kelly Newton, Sophie S. C. Leterme, Ben Roudnew, Renee J. Smith, Laurent Seuront, James G. Mitchell

**Affiliations:** 1 School of Biological Sciences, Flinders University, Adelaide, South Australia, Australia; 2 Plant Functional Biology and Climate Change Cluster, University of Technology Sydney, Sydney, Australia; 3 Plymouth Marine Laboratory, Plymouth, United Kingdom; 4 Institute of Genomic and Systems Biology and Department of Biosciences, Argonne National Laboratory, Argonne, Illinois, United States of America; 5 Department of Ecology and Evolution, University of Chicago, Chicago, Illinois, United States of America; 6 Department of Biology, San Diego State University, San Diego, California, United States of America; 7 Aquatic Sciences, South Australian Research and Development Institute, Henley Beach, South Australia, Australia; 8 Centre National de la Recherche Scientifique, Paris, France; Université Paris Sud, France

## Abstract

Environmental parameters drive phenotypic and genotypic frequency variations in microbial communities and thus control the extent and structure of microbial diversity. We tested the extent to which microbial community composition changes are controlled by shifting physiochemical properties within a hypersaline lagoon. We sequenced four sediment metagenomes from the Coorong, South Australia from samples which varied in salinity by 99 Practical Salinity Units (PSU), an order of magnitude in ammonia concentration and two orders of magnitude in microbial abundance. Despite the marked divergence in environmental parameters observed between samples, hierarchical clustering of taxonomic and metabolic profiles of these metagenomes showed striking similarity between the samples (>89%). Comparison of these profiles to those derived from a wide variety of publically available datasets demonstrated that the Coorong sediment metagenomes were similar to other sediment, soil, biofilm and microbial mat samples regardless of salinity (>85% similarity). Overall, clustering of solid substrate and water metagenomes into discrete similarity groups based on functional potential indicated that the dichotomy between water and solid matrices is a fundamental determinant of community microbial metabolism that is not masked by salinity, nutrient concentration or microbial abundance.

## Introduction

Microbes numerically dominate the biosphere and play crucial roles in maintaining ecosystem function by driving chemical cycles and primary productivity [1], [2]. They represent the largest reservoir of genetic diversity on Earth, with the number of microbial species inhabiting terrestrial and aquatic environments estimated to be at least in the millions [3]. However, the factors determining the spatiotemporal distributions of microbial species and genes in the environment are only vaguely understood, but are likely to include micro-scale to global-scale phenomena with different controlling elements.

Microbial community structure is determined on varying scales by a complex combination of historical factors (e.g. dispersal limitation and past environmental conditions) [4], the overall habitat characteristics [5], the physical structure of the habitat (e.g. fluid or sediment) and by changes in current environmental parameters (e.g. salinity and pH) [6]–[9]. Understanding the relative importance of these different effectors is central to understanding the role of microbes in ecosystem function, and therefore to predicting how resident microbial communities will adapt to, for example, increasing salinity levels due to localized climate driven evaporation and reduced rainfall [10].

Physicochemical gradients provide natural model systems for investigating the influence of environmental variables on microbial community structure. In aquatic systems, salinity is a core factor influencing microbial distribution [6], [11] and has been identified as the primary factor influencing the global spatial distribution of microbial taxa [6]. Salinity gradients occur in estuaries, solar salterns and ocean depth profiles. Evidence exists for increases in abundance and decreases in the diversity of microbial communities spanning salinity gradients [9], [11]–[14]. This change is wrought by variance in the halo-tolerance of different taxa and the influence of salinity on nutrient concentrations [15].

We examined the resident microbial communities inhabiting sediment at four points along a continuous natural salinity gradient in the Coorong, a temperate coastal lagoon located at the mouth of the Murray River, South Australia. To determine the relative importance of salinity, nutrient status and microbial abundance in structuring microbial community composition and function, we used shotgun metagenomics to compare the taxonomic and metabolic profiles of our samples to representative metagenomes in public databases. Our results demonstrate that the taxonomic composition and metabolic potential of our metagenomes show a conserved signature, despite the microbes existing in disparate chemical environments. Comparison to other metagenomes indicates that this signature is determined by the substrate type (i.e. sediment) of the samples.

## Results

### Biogeochemical environment

Dramatic shifts in physiochemical conditions occurred across the Coorong lagoon, with salinity notably varying from 37 to 136 practical salinity units (PSU) and inorganic nutrient levels changing by over an order of magnitude between sampling locations (Table 1). Practical Salinity Units (PSU) are the standard measurement of salinity in oceanography and represent a ratio of the conductivity of a solution relative to a standard, and is approximately convertible to parts per thousand of salt. For context seawater has an average salinity of 35 PSU [16]. Additionally, the abundance of heterotrophic bacteria and viruses, as determined by flow cytometry [17], [18], increased along the salinity gradient by 31 fold and 28 fold respectively. The microbial community inhabiting this environmental gradient was explored using metagenomics, where microbial DNA was extracted and sequenced from each sampling site using a 454 GS-FLX platform (Roche). The sampling yielded between 16 Mbp and 27 Mbp of sequence information per library (Table 1). Approximately 30% of the sequences from each library had significant (BLASTX E-value<10^−5^) matches to the SEED non-redundant database [19] as determined using the MetaGenomics Rapid Annotation using Subsystem Technology (MG-RAST) pipeline [20].

**Table 1:** Sequencing data and environmental metadata for metagenomic sampling sites.

Sampling Site	37 PSU	109 PSU	132 PSU	136 PSU
Number of reads	68888	101003	114335	108257
Average read length (bp)	232	234	232	232
% Sequences matching SEED subsystems	27	30	26	29
Salinity (PSU)	37	109	132	136
pH	8.25	7.85	7.79	8.05
Temperature (°C)	21	25	27	24
Ammonia concentration (mgN/L)	0.23 (±0.15)	0.21 (±0.09)	0.96 (±0.31)	3.10 (±0.84)
Phosphate concentration (mgP/L)	0.05 (±0.01)	0.11 (±0.02)	0.12 (±0.03)	0.27 (±0.09)
Porewater bacteria concentration (per mL)	4.8×10^6^ (±6.3×10^5^)	7.4×10^7^ (±8.4×10^6^)	7.2×10^7^ (±4.2×10^6^)	1.5×10^8^ (±1.4×10^7^)
Porewater virus concentration (per mL)	1.5×10^7^ (±5.8×10^6^)	2.3×10^8^ (±3.1×10^7^)	1.8×10^8^ (±1.5×10^7^)	4.2×10^8^ (±3.1×10^7^)
Turbidity of water column (NTU)	7	16	16	10
Dissolved Oxygen in water column (%)	93	140	134	89

Percentage of sequences matching SEED subsystems were determined with an E-value cutoff of E<1×10^−5^. All metadata was measured in sediment interstitial porewater with the exception of turbidity and dissolved oxygen which were measured in the overlying water column. ± indicates Standard error of the mean (n = 3 for nutrient measures, n = 5 for microbial abundances). N = nitrogen, P = phosphate, PSU = practical salinity units, NTU = Nephelometric Turbidity Units.

### Taxonomic and metabolic profiling of metagenomes along an environmental gradient

**Figure 1:**
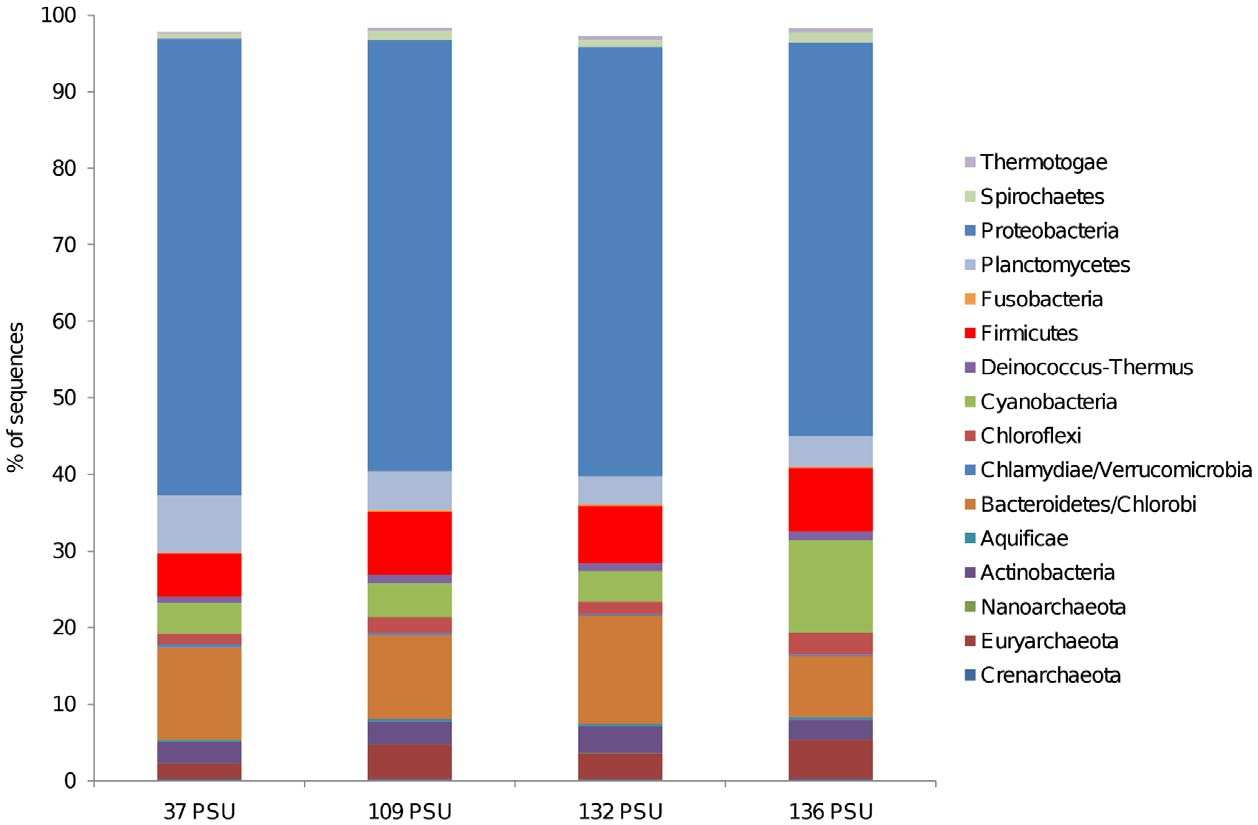
Taxonomic composition (Phyla level) of four metagenomic libraries derived from Coorong lagoon sediment. Relative representation in the metagenome was calculated by dividing the number of hits to each category by the total number of hits to all categories, thus normalizing by sequencing effort. Hits were generated by BLASTing sequences to the SEED database with an E-value cut-off of 1×10^−5^ and a minimum alignment of 50 bp.

All metagenomic libraries were dominated by bacteria (94% of hits to the SEED database) with sequences also matching the archaea (4%), eukarya (1.5%) and viruses (0.2%). The bacterial phylum, *Proteobacteria*, dominated all four metagenomic libraries, representing over 50% of taxonomic matches for SEED taxonomy (Fig. 1) and over 40% of ribosomal DNA matches (Table S1). Other prominent phyla included the *Bacteroidetes/Chlorobi* group (approx. 8–14%), *Firmicutes* (approx. 6–8%), and *Planctomycetes* (approx. 4–7%). In the metagenome from the 136 PSU environment, *Cyanobacteria* were the second most represented phylum, representing approximately 12% of the community, in the metagenomic datasets (Fig. 1) but were less prominent in the other samples, representing approximately 4%. In the ribosomal DNA profiles generated from BLAST matches of metagenome sequences against the Ribosomal Database Project [21] (Table S1), *Cyanobacteria* were the second most abundant classified phylum in both the 132 PSU and 136 PSU metagenomes. At the phylum level, profiles were highly conserved between the four samples (Fig. 1). At level 3 within the MG-RAST hierarchical classification scheme, which includes orders and classes [20], the most abundant taxa in all four metagenomes were the classes *γ-proteobacteria* and *α-proteobacteria* which represented approximately 20% of sequence matches. *Cyanobacteria* in the 136 PSU metagenome were predominantly represented by the orders *Nostocales* (order) and *Chroococcales*, which each comprised approximately 40% of cyanobacterial hits (Table S2).

**Figure 2:**
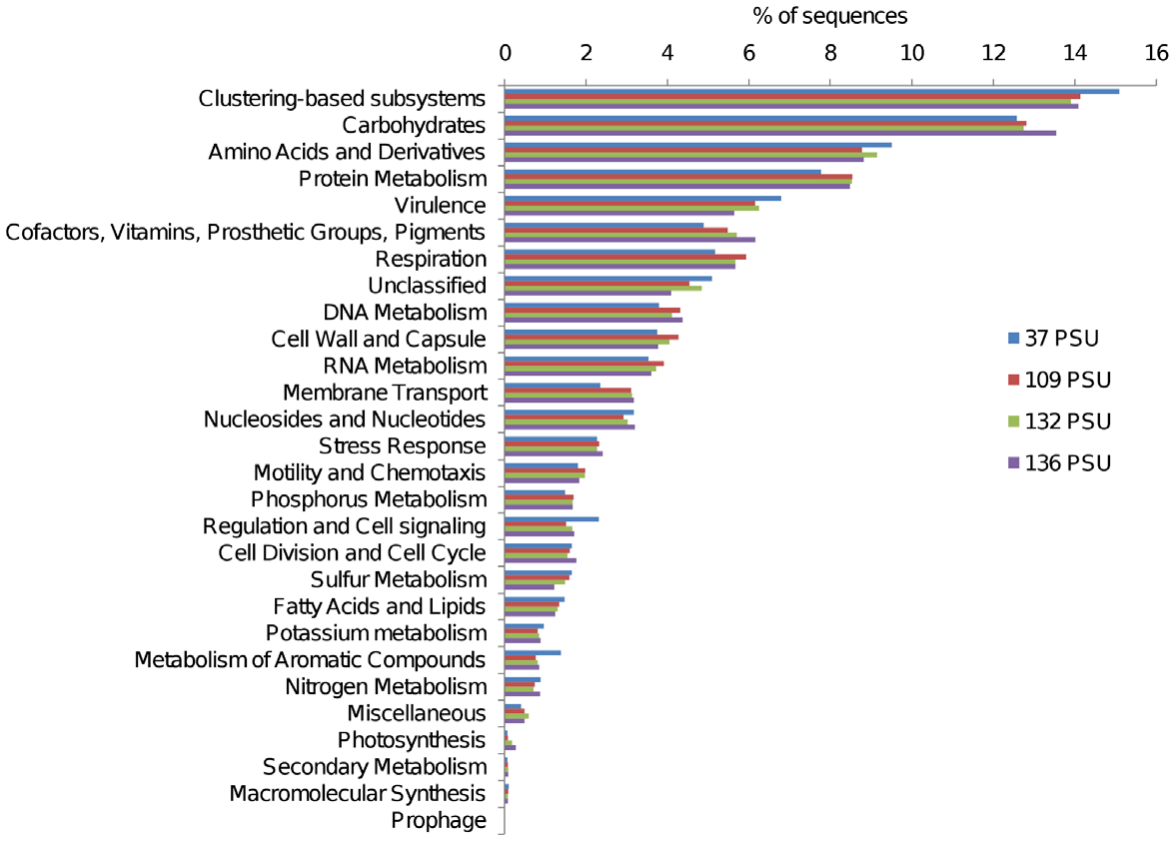
Metabolic composition of four metagenomic libraries derived from Coorong lagoon sediment. Relative representation in the metagenome was calculated by dividing the number of hits to each category by the total number of hits to all categories, thus normalizing by sequencing effort. Hits were generated by BLASTing sequences to the SEED database with an E-value cut-off of 1×10^−5^ and a minimum alignment of 50 bp.

**Figure 3:**
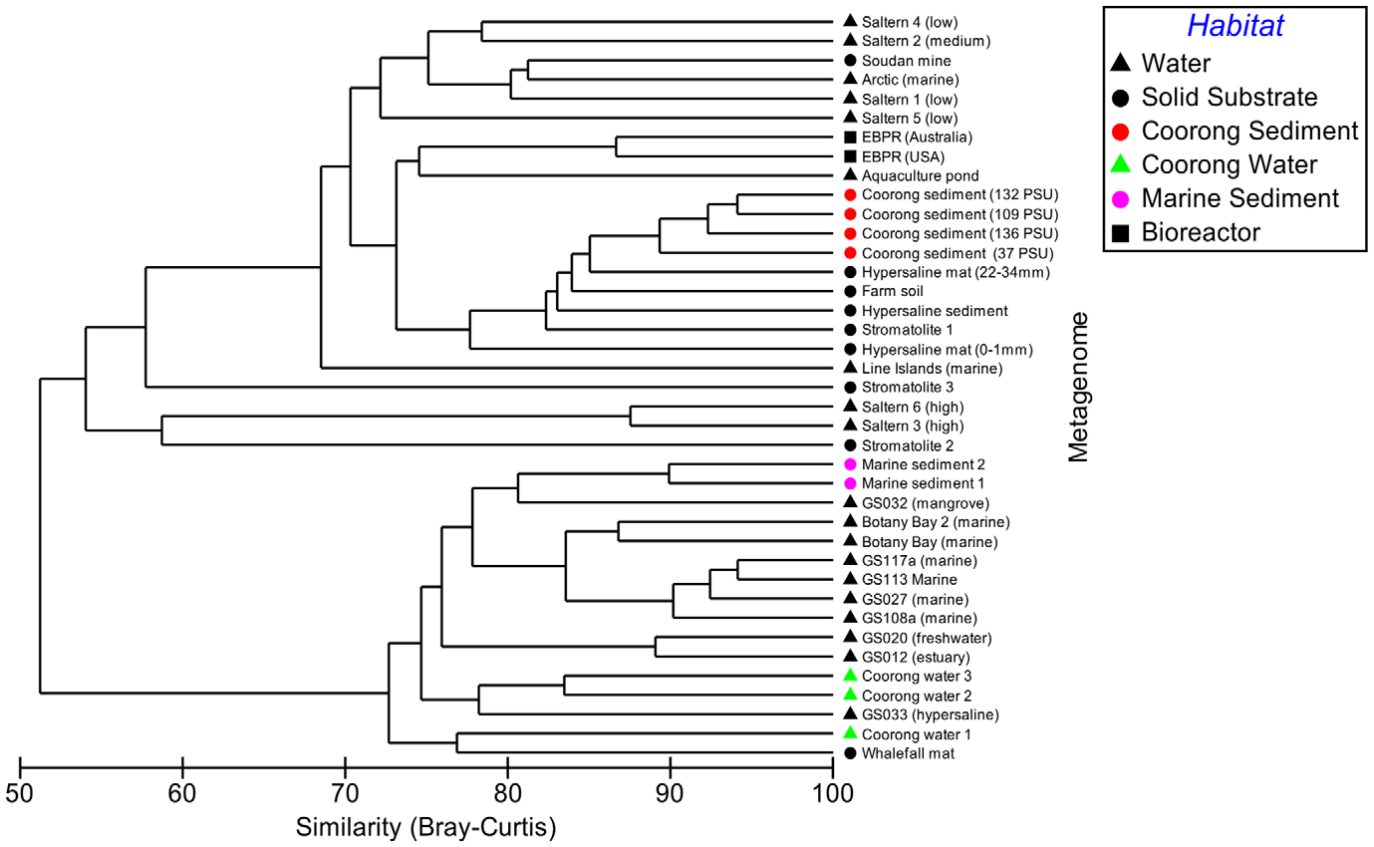
Comparison of taxonomic profiles derived from selected metagenomes publicly available on the MG-RAST database. The hierarchical agglomerative cluster plot (group average) is derived from a Bray-Curtis similarity matrix calculated from the square root transformed abundance of DNA fragments matching taxa in the SEED database (BLASTX E-value<0.001, genome level taxonomy).

**Figure 4:**
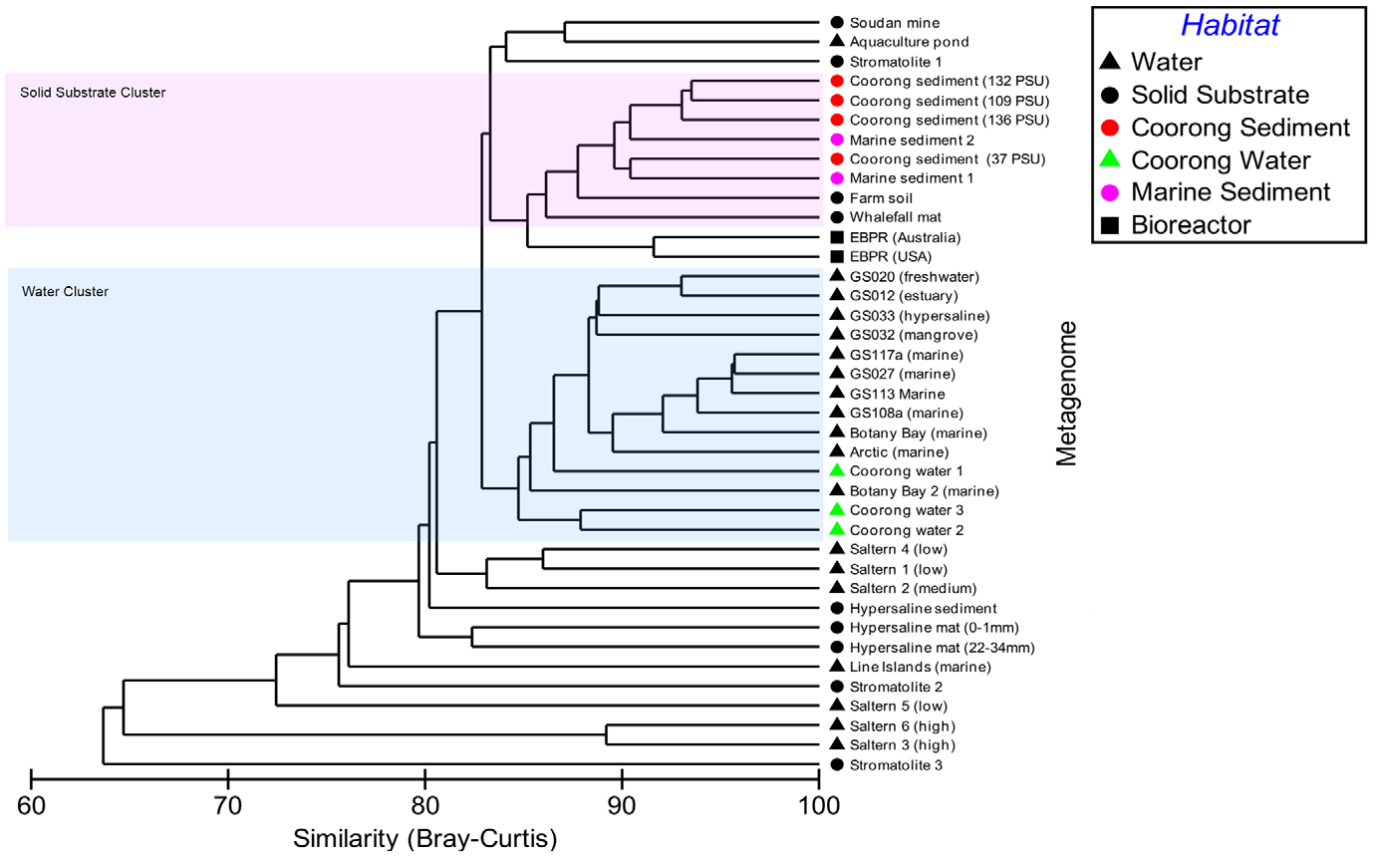
Comparison of metabolic profiles derived from selected metagenomes publicly available on the MG-RAST database. The hierarchical agglomerative cluster plot (group average) is derived from a Bray-Curtis similarity matrix calculated from the square root transformed abundance of DNA fragments matching subsystems in the SEED database (BLASTX E-value<0.001).

All Coorong metagenomes were dominated by the core metabolic functions of carbohydrate, amino acid and protein metabolism. Metabolisms indicative of a functionally diverse community were represented with heterotrophic nutrition, photosynthesis, nitrogen metabolism and sulfur metabolism contributing to the profile (Fig. 2). Paralleling the pattern observed for the taxonomic profiles, metabolic profiles were conserved between the four samples in terms of broadly defined metabolic processes, classified at the coarsest level of functional hierarchy within the MG-RAST database (Fig. 2). Metagenomic profiles remained highly conserved at the genome level, which we used to compare the Coorong metagenomes to each other and to other metagenomes from diverse habitats (Fig. 3), and at the level of individual cellular processes, termed subsystems, which is the finest level of metabolic hierarchy within the MG-RAST database [20] (Fig. 4).

### Comparison to metagenomic profiles from other habitats

We compared the taxonomic and metabolic structures of our metagenomes to those from a wide variety of habitats, including other hypersaline and marine sediment environments (Table 2, Table S3), using high resolution profiles derived at the genome and metabolic subsystem [19] level. For both taxonomic and metabolic profiles (Figs. 3 & 4), Coorong metagenomes showed a high degree of statistical similarity (Bray-Curtis) to each other, despite the strong habitat gradients from which they were derived. Taxonomically, our metagenomes were all >89% similar with the 136 PSU sample diverging at 92% similarity from the 109 PSU and 132 PSU profiles which were 94% similar. In terms of metabolic potential, they were >89.5% similar with the 136 PSU sample diverging at 93% similarity from the 109 PSU and 132 PSU profiles which were 93.5% similar.

**Table 2:** Summary of metagenomes used in this study.

MG-RAST ID	Description/Reference	MG-RAST ID	Description/Reference
4440984.3	**Coorong sediment (37 PSU)**	4440971.3	Hypersaline mat (22–34 mm) [29]
4441020.3	**Coorong sediment (109 PSU)**	4441584.3	GS012 (Estuary) [45]
4441021.3	**Coorong sediment (132 PSU)**	4441590.3	GS020 (freshwater) [45]
4441022.3	**Coorong sediment (136 PSU)**	4441595.3	GS027 (Marine) [45]
4446406.3	Coorong water 1	4441598.3	GS032 (mangrove) [45]
4446412.3	Coorong water 2	4441599.3	GS033 (hypersaline) [45]
4446411.3	Coorong water 3	4441606.3	GS108a (marine) [45]
4446341.3	Marine sediment 1	4441610.3	GS113 (marine) [45]
4446342.3	Marine sediment 2	4441613.3	GS117a (marine) [45]
4440329.3	Hypersaline sediment	4443688.3	Botany Bay (marine)
4440324.3	Saltern 1 (low) [5], [28]	4443689.3	Botany Bay 2 (marine)
4440435.3	Saltern 2 (medium) [5], [28]	4440041.3	Line Islands (marine) [46]
4440438.3	Saltern 3 (high) [5], [28]	4440212.3	Arctic (marine) [47]
4440437.3	Saltern 4 (low) [5], [28]	4440440.3	Aquaculture pond [5]
4440426.3	Saltern 5 (low) [5], [28]	4440281.3	Soudan mine [48]
4440429.3	Saltern 6 (high) [5], [28]	4441656.4	Whalefall mat [49]
4440067.3	Stromatolite 1 [50]	4441093.3	EBPR (USA) [51]
4440060.4	Stromatolite 2 [50]	4441092.3	EBPR (Australia) [51]
4440061.3	Stromatolite 3 [5]	4441091.3	Farm soil [49]
4440964.3	Hypersaline mat (0–1 mm) [29]		

All metagenomes are publicly available on the MG-RAST server (http://metagenomics.nmpdr.org/) [20]. Number of database hits (BLASTX) are determined using an E-value cut-off of 0.001. A more detailed table is provided in supporting information Table S3. Bold = this study.

The metagenomes which exhibited the greatest taxonomic similarity to the Coorong samples were from a hypersaline microbial mat, farm soil, hypersaline sediment and a freshwater stromatolite. These samples formed a discrete cluster of >82% similarity in our hierarchical tree (Fig. 3). Those with the greatest metabolic similarity to the Coorong samples were from marine sediment, farm soil, phosphorous removing sludge and a whalefall microbial mat. These samples formed a discrete cluster of >85% similarity in our hierarchical tree (Fig. 4). Notably, these metagenomes were all derived from sediment, soil, biofilm or mat samples (termed ‘solid substrate’ in this study) and particle rich bioreactor sludge, but varied in salinity from non-saline to hypersaline. Hypersaline water samples from the Coorong lagoon (Newton *et al*, in prep), with similar salinities to our data, did not cluster with the Coorong sediment metagenomes in terms of taxonomy or metabolism, but rather clustered with water samples from a variety of other habitats. Marine sediment samples however, clustered with the Coorong sediment metagenomes for metabolic but not taxonomic profiles. Overall, solid substrate and water metagenomes clustered into discrete metabolic similarity groups with nodes of 85% similarity.

## Discussion

Despite the strong environmental heterogeneity along the gradient studied here (Table 1), taxonomic and metabolic profiles were conserved at the phyla and SEED hierarchy 1 level (Figs. 1 & 2). This similarity was even more striking at finer levels of resolution. Coorong metagenome profiles were >89% and 89.5% similar in taxonomic and metabolic composition at the genome and subsystem level respectively (Figs. 3 & 4). This indicates that the four microbial communities had similar structure, despite the intense environmental variability that occurred along the gradient. While the strong similarity between these samples, relative to other samples of comparable salinity, may to some extent be attributable to identical DNA extraction and sequencing procedures, biogeography and a shared environmental history between the samples, the clustering of our metagenomes with other solid substrate metagenomes for both taxonomic and metabolic profiles at >82% and >85% respectively, indicates that the signature of our profiles is largely determined by the substrate type of the samples (i.e. sediment). The metagenomes which show a high degree of similarity to our profiles are derived from a wide range of salinities, indicating that salinity is not the major structuring factor.

Particularly evident is the close metabolic clustering of the four Coorong sediment metagenomes with other examples of marine sediment (Fig. 4) despite these samples coming from a lower salinity than the Coorong sediment samples. This principle is highlighted by the observation that Coorong water samples of a similar salinity and identical geographic location (Table S3) do not cluster with Coorong sediment samples in terms of taxonomy or metabolic potential, but rather cluster with other water samples. We interpret this as an indication that the substrate type (e.g. water vs solid substrate) is an important determinant of microbial functional composition that supersedes bulk environmental parameters (e.g. salinity) as the dominant structuring factor. This is further supported by the observation that the majority of metagenomes analyzed for metabolic potential cluster into two groups: a water group and a solid substrate group (Fig. 4), regardless of salinity or geographic location. Whilst it has been shown that metagenomic profiles cluster into defined biome groups [5], [22], this is the first observation of such a clear dichotomy between water and solid substrate habitats which is not masked by salinity.

Salinity has previously been identified as the primary factor governing the global distribution of prokaryotic 16S rRNA sequences [6], [23], [24], [25]. Whilst Lozupone & Knight [6] identified substrate type (water vs sediment) as the second most important factor structuring microbial diversity after salinity, Tamames *et al* [24] concluded that salinity is more relevant than substrate type as sediment/soil and water from similar salinities clustered together in their analysis. These findings contradict the patterns apparent in our metabolic profile clustering (Fig. 4) and indicate that the phylogenetic and metabolic aspects of microbial community diversity may be driven by different dominant factors. This also implies that accessing genetic information from the entire length of the genome as opposed to a specific taxonomic marker gene can yield different interpretations. This is potentially due to the influence of lateral gene transfer and a wider representation of taxa in 16S rDNA databases as opposed to genomic databases [26], [27]. Whilst Coorong metagenomes clustered taxonomically with other solid substrate metagenomes (Fig. 3), there was not a clear dichotomy between samples from water and solid substrate types as was observed for the metabolic profiles. This indicates that the substrate type may not be as important a controlling factor for taxonomy as it is for metabolism. That substrate type is a more important determinant of metabolic composition indicates that some genes, important for living in different substrate types, are shared by varying taxa adapted to different salinities.

The samples that did not metabolically cluster within the two larger branches of ‘solid substrate’ and water (Fig. 4) were typically derived from more extreme hypersaline environments, such as solar salterns [28] and a hypersaline mat [29]. This indicates that in some cases, salinity can be the major factor driving the metabolic profile grouping, probably in instances where salinity reaches a critical level, whereby it selects for less diversity and more dominant taxa. This is consistent with the salinity driven clustering of the saltern metagenomes when ordinated using di-nucleotide signatures [22].

The characteristics of particular substrate types that can select the metabolic content of the microbial community could be related to the differing degree of chemical heterogeneity in fluid and solid substrate habitats. Water is mixed to a higher degree than soil/sediment thus resulting in less physiochemical heterogeneity. Soil, sediment and biofilms are extremely heterogeneous resulting in the high degree of diversity commonly observed in these habitats compared to water substrates [3], [6]. This differing division of resources and niches likely explains the dichotomous clustering of water and solid substrate metagenomes observed in our data. Additionally, in aquatic systems, sediment and benthic habitats are generally more anoxic than the overlying water suggesting that reduction and oxidation (REDOX) status is also a potentially important factor driving this split. Indeed, initial investigations indicate that a prevalence of virulence, motility and anaerobic respiration genes in solid substrate habitats drive the water versus solid substrate split (Jeffries *et al*, in prep).

Our interpretation that the matrix from which the sample is derived is more important in determining the functional community structure than bulk physicochemical conditions has important implications for how we predict changes in microbial community function in the context of climate change driven increases in salinity levels or eutrophication associated with anthropogenic inputs. For example, the Coorong is currently undergoing a period of increasing salinity levels and eutrophication [30], reflected in the gradient examined here. Our results suggest that, whilst small scale changes in gene abundance occur across this salinity gradient (for example regulation/signaling and metabolism of aromatic compounds; Fig. 2), the overall functional potential of the microbial community remains similar between salinities and demonstrates a high degree of similarity to lower salinity marine sediment at the subsystem level (Fig. 4). This indicates that while shifts in the composition of the microbial community may occur following further shifts in salinity, the overall biogeochemical potential of the community may remain relatively unchanged. Of course, extreme increases in salinity will potentially result in the emergence of dominant specialist species, decreasing diversity and potentially influencing function.

There is the potential that the discrete clustering of our samples may be related to technical bias, because of the different strategies for sample collection, sequencing and analysis of metagenomes from other locations. However, when we compared our data with metagenomes generated using different DNA extraction techniques and sequencing platforms, no discernible pattern emerged that can link the relatedness of metagenomes to elements of methodology (Figs. 3 & 4). DNA extraction and sequencing techniques have also been shown not to significantly influence metagenomic profile discrimination by habitat [31]. Additionally, marine sediment samples extracted in the same lab using identical techniques did not cluster taxonomically with the Coorong samples (Fig. 3) and Coorong water samples extracted using the same lab and techniques did not cluster with the Coorong sediment samples (Figs. 3 & 4), indicating methodology is not obscuring environmental clustering. One caveat that should be considered when interpreting our data is the use of annotated data to compare metagenomes. Our data is reflective of the genomes and metabolic subsystems present in the MG-RAST database [20] and should be interpreted as patterns observed in the context of this diversity. Metagenomic databases are composed of taxa for which whole genome sequences exist, which represent a biased subsection of microbial diversity heavily skewed towards cultured organisms chosen because of ease of growth or interesting phenotypes [26], [27]. Thus the databases tend to be skewed towards the phyla *Proteobacteria*, *Firmicutes*, *Actinobacteria* and *Bacteroidetes* [26]. Whilst genome based databases represent a valid reference point for relative comparison of the taxonomic affiliation of subsystems observed in the data, which has been routinely applied for metagenomes [20] a much broader view of the taxonomic variability can be provided by the 16S rDNA gene [26]. Further analysis using clustering algorithms [32] and di-nucleotide frequencies [22] will shed light on how our un-annotated data is similar to other metagenomes.

This study focused on the balance between taxonomic and metabolic identifiers to determine the dominant controlling environmental factor. We found substrate type is the dominant controller of gene abundance. To date, the majority of community scale microbial biogeography studies have considered the presence or absence of particular taxonomic units. In many cases however, microbial biogeography is not binary, with most taxa being present but at a low abundance in the so called ‘rare biosphere’ [33]. Additionally, functional genes may be passed between different taxa via lateral gene transfer [34], [35] indicating that taxonomy alone is not a determinant of community function. More sophisticated approaches which consider complex patterns in the metagenomic structure of communities and the complex interactions between different drivers acting on different scales are necessary to understand the spatial distribution of microbial diversity. High throughput sequencing allows profiling of both taxonomic and metabolic diversity and when coupled to statistical techniques [5], [36]–[39] and standardized records of metadata [40] patterns in the composition of microbial metagenomes begin to emerge. One such pattern in our data is the high degree of taxonomic and functional similarity between metagenomes derived across a strong salinity, nutrient and abundance gradient and between metagenomes derived from sediment/soil/mat metagenomes regardless of salinity. Another pattern is the dichotomous clustering of solid substrate metagenomes and water metagenomes into discrete similarity groups which are not masked by differences in salinity. Overall our results suggest that substrate type (water or solid substrate) plays a fundamental role in determining the composition of the metagenome and that, in addition to extant physiochemical parameters, needs to be considered when interpreting patterns in microbial community diversity.

## Materials and Methods

### Site selection and sediment sampling

Sampling was conducted along the 100 km long, shallow temperate coastal lagoon comprising the Coorong, in South Australia (35°33′3.05″S, 138°52′58.80″E), which is characterized by a strong continuous gradient from estuarine to hypersaline salinities. Samples were collected from four sites along the salinity gradient. The sites were characterized by differing salinities and nutrient status (Table 1). Sediment for DNA extraction was sampled using a new 1.5 cm diameter sterile corer at each site, and included the upper 10 cm of sediment. Sample cores were transferred to a sterile 50 mL centrifuge tube, stored and transported on ice in the dark following collection, and DNA extraction was undertaken within six hours of sampling.

For each site, nutrient levels in porewater and overlying water were determined using a Lachat QuikChem 8500 nutrient analyzer and pH, dissolved oxygen and salinity were measured using a 90FL-T (TPS) multi-parameter probe. Abundance of heterotrophic bacteria and viruses in sediment porewater was assessed using a Becton Dickinson FACScanto flow cytometer and previously described protocols [17], [18]. In line with previous studies (e.g. [41]), porewater microbial abundance was used to compare sediment samples using flow cytometry, potentially representing a lower estimate of the entire sediment abundance [42], which includes particle-attached bacteria and viruses. Sampling was conducted under a Government of South Australia Department of Environment and Heritage Permit to Undertake Scientific Research.

### Metagenomic sequencing

Microbial community DNA was extracted from c.a.10 g of homogenized sediment, using the entire volume of the sediment core, using a bead beating and chemical lysis extraction kit (MoBio, Solano Beach, CA.) and further concentrated using ethanol precipitation. DNA quality and concentration was determined by agarose gel electrophoresis and spectrophotometry and >5 µg of high molecular weight DNA was sequenced at the Australian Genome Research Facility. Sequencing was conducted on a GS-FLX pyrosequencing platform (Roche) using a multiplex barcoding approach to distinguish between the four libraries on a single plate. Sequencing yielded between 16 Mbp and 27 Mbp of sequence information per library, with an average read length of 232.5 bp (Table 1).

### Bioinformatics and statistical analysis

Unassembled sequences (environmental gene tags) were annotated using the MetaGenomics Rapid Annotation using Subsystem Technology (MG-RAST) pipeline version 2.0 (http://metagenomics.nmpdr.org/) [20], with a BLASTX E-value cut-off of E<1×10^−5^ and a minimum alignment length of 50 bp. The abundance of individual sequences matching a particular SEED subsystem (groups of genes involved in a particular metabolic function) [19] were normalized by sequencing effort and used to generate a metabolic profile of the metagenome. Taxonomic profiles were generated within MG-RAST using the normalized abundance of the phylogenetic identity of sequence matches to the SEED database [19] and Ribosomal Database Project (Table S1) both with a BLAST E-value cut-off of E<1×10^−5^ and a minimum alignment length of 50 bp [21]. The MG-RAST pipeline [20] implements the automated BLASTX annotation of metagenomic sequencing reads against the SEED non-redundant database [19], a manually curated collection of genome project derived genes grouped into specific metabolic processes termed ‘subsystems’. The SEED matches of Protein Encoding Genes (PEGs) derived from the sampled metagenome may be reconstructed either in terms of metabolic function or taxonomic identity at varying hierarchical levels of organization. For taxonomy, there are five levels from domain to genome level and for metabolism there are three sequential nested groupings termed level 1, level 2 and subsystem. In our data, metabolic information was derived at the coarsest level of organization, the generalized cellular functions, termed level 1 (Fig. 2), and the finest, individual subsystems (Fig. 4). Taxonomy was profiled at the phylum (Fig. 1) and genome (Fig. 3) level. In order to statistically investigate the similarity of the four Coorong metagenomes, as well as the metagenomic profiles publicly available on the MG-RAST server and in our own database (Table 2, Table S3), we generated a heatmap of the frequency of MG-RAST hits to each individual taxa (genome level) or subsystem for each metagenome, which had been normalized by dividing by the total number of hits to remove bias in sequencing effort or differences in read length. These hits were identified using an E-value cut-off of E<0.001. Statistical analyses were conducted on square root transformed frequency data using Primer 6 for Windows (Version 6.1.6, Primer-E Ltd. Plymouth) [43]. Hierarchical agglomerative clustering (CLUSTER) [44] was used to display the Bray-Curtis similarity relationships between our profiles and those of the publicly available metagenomes with the results displayed as a group average dendogram. Specific Bray-Curtis similarities for individual clusters were taken from the Primer 6 CLUSTER output, which displays the stepwise construction of the dendogram.

## Supporting Information

Table S1 **Percentage of Ribosomal DNA matches to bacterial phyla.** Relative representation in the metagenome was calculated by dividing the number of hits to each category by the total number of hits to all categories. Hits were generated by BLASTing sequences to the Ribosomal Database Project [21], via MG-RAST [20], with an E-value cut-off of 1×10^−5^ and a minimum alignment of 50 bp. Due to inconsistencies in 16S rDNA copy number, these relative abundances represent estimates of overall ribosomal DNA composition at phyla level only.
(DOC)

Table S2 **Relative proportion of matches to the SEED taxonomic hierarchy.** Relative representation in the metagenome was calculated by dividing the number of hits to each category by the total number of hits to all categories. Hits were generated by BLASTing sequences to the SEED database with an E-value cut-off of 1×10^−5^ and a minimum alignment of 50 bp.
(XLS)

Table S3 **Detailed summary of metagenomes used in this study.** All metagenomes are publicly available on the MG-RAST server (http://metagenomics.nmpdr.org/) [20]. Number of database hits (BLASTX) are determined using an E-value cut-off of 0.001. References are provided in Table 2 of the manuscript. Bold = this study.
(XLS)
