# Supplementary material for: Substrate Type Determines Metagenomic Profiles from Diverse Chemical Habitats
Source: PLoS One. 2011 Sep 23;6(9):e25173. doi: 10.1371/journal.pone.0025173 (PMC3179486; doi:10.1371/journal.pone.0025173)
Supplement: Table S1 — Percentage of Ribosomal DNA matches to bacterial phyla. Relative representation in the metagenome was calculated by dividing the number of hits to each category by the total number of hits to all categories. Hits were generated by BLASTing sequences to the Ribosomal Database Project [21], via MG-RAST [20], with an E-value cut-off of 1×10^−5^ and a minimum alignment of 50 bp. Due to inconsistencies in 16S rDNA copy number, these relative abundances represent estimates of overall ribosomal DNA composition at phyla level only. (DOC) [file pone.0025173.s001.doc]

| PHYLUM | 37 PSU | 109 PSU | 132 PSU | 136 PSU |
| --- | --- | --- | --- | --- |
| Actinobacteria | 11.43 | 8.11 | 2.78 | 4.82 |
| Aquificae | 0 | 0 | 0 | 4.82 |
| Bacteroidetes | 8.57 | 12.16 | 12.5 | 6.02 |
| Cyanobacteria | 5.71 | 0 | 16.67 | 12.05 |
| Firmicutes | 0 | 5.41 | 4.17 | 3.61 |
| Genera_incertae_sedis_WS3 | 0 | 4.05 | 0 | 0 |
| Planctomycetes | 0 | 2.7 | 1.39 | 0 |
| Proteobacteria | 57.14 | 47.3 | 40.28 | 45.78 |
| Spirochaetes | 0 | 5.41 | 8.33 | 4.82 |
| unclassified_Bacteria | 25.71 | 24.32 | 27.78 | 32.53 |
